# Why we need to better understand the cortical neurophysiology of impaired postural responses with age, disease, or injury

**DOI:** 10.3389/fnint.2014.00069

**Published:** 2014-08-29

**Authors:** Jesse V. Jacobs

**Affiliations:** Human Motion Analysis Laboratory, Department of Rehabilitation and Movement Science, University of VermontBurlington, VT, USA

**Keywords:** automatic postural responses, balance, cortex, Parkinson's disease, low back pain, aging, stroke

## Introduction and purpose

The ability to maintain standing balance and orientation is crucial to mobility and independence. Standing balance is generally maintained by anticipatory postural adjustments associated with voluntary actions. However, inaccurate judgment or impaired anticipatory processes, as well as extrinsic postural perturbations–external forces not generated intrinsically due to voluntary movement (e.g., due to a slip, trip, push, etc.)–can render the need for reactive postural control to recover orientation and balance (Fasano et al., 2012).

For people with disorders of posture and balance associated with aging, neurodegeneration, or injury, an appropriately timed and coordinated postural response to extrinsically perturbed balance may represent the crucial difference between a harmless balance recovery and an aggravated pain condition or injurious fall. Indeed, postural responses to slips and sudden changes in load are common circumstances for incurring an episode of low back pain (LBP), a worldwide leading cause of disability (Manning et al., 1984; Andersson, 1999). In addition, falls associated with aging or neurological disorders are leading causes of injury, decreased activity participation, morbidity, and mortality (Grimbergen et al., 2004; Finlayson and Peterson, 2010; Batchelor et al., 2012). For these reasons research must detail the neurophysiology responsible for producing both healthy and impaired human postural responses to an extrinsically induced perturbation of standing balance.

A recently proposed neurophysiologic model of extrinsically induced postural responses reviewed initial evidence that the cerebral cortex influences these postural responses by (a) priming the most contextually accurate response during preparation, and (b) modifying late response phases (Jacobs and Horak, 2007). The involvement of the cerebral cortex during early response phases thus appears indirect and limited to priming sub-cortically generated synergies based on contextual features known prior to the perturbation, but the cortex can then directly participate in modifying the late response phases to improve response efficacy. Cortical functions associated with priming contextually appropriate responses are thus represented through pre-perturbation measures of cortical activity such as pre-movement potentials, and cortical functions associated with online modifications to late-phase responses are represented by measures of cortical activity following perturbation onset, such as perturbation evoked potentials (PEPs) (Adkin et al., 2008; Jacobs et al., 2008; Mochizuki et al., 2008, 2010). Further research remains needed, however, due to a paucity of data on neural mechanisms of human postural responses with disease or injury.

Understanding cortical function associated with impaired postural responses is essential given (a) the important role of the cerebral cortex for generating postural responses, (b) the potential that its influence may be enhanced to compensate for impaired automated processes of sub-cortical control, and (c) the accessibility of cortex for neuroplastic change with intervention. Unfortunately, because postural responses to extrinsic perturbations were historically considered reflexive and indicative of sub-cortical processing, the literature regarding cortical influence on extrinsically induced postural responses is not well developed. Whereas a more longstanding and developed literature exists on the role of the cerebral cortex for generating anticipatory postural adjustments with voluntary movement—both in the use of many methods of neurophysiologic recording and in the evaluation of people with health conditions (Gurfinkel and El'ner, 1988; Massion, 1992; Saitou et al., 1996; MacKinnon et al., 2007; Tsao et al., 2008; Jacobs et al., 2009a, 2010; Ng et al., 2012; Lomond et al., 2013; Papegaaij et al., 2014), the literature on postural responses to extrinsically induced perturbations is less extensive.

Therefore, the purpose of this opinion article is to focus on the cortical neurophysiology of impaired human postural responses to extrinsic perturbations of upright stance. This article will highlight the insights provided from rare studies of cortical function in people with impaired standing postural responses in order to demonstrate the need and potential value of future research focused on the cortical neurophysiology of impaired human postural responses to extrinsic perturbations.

## What we can learn from cortical neurophysiology during impaired postural responses

Table 1 highlights common movement impairments and our knowledge of cortical neurophysiology associated with human postural responses to perturbations of standing balance for selected example conditions of stroke, advanced age, Parkinson's disease (PD), and LBP. To demonstrate the case for this opinion article, these conditions represent samples of a much larger scope of postural disorders related to cortical injury, age- and disease-associated neurodegeneration, as well as chronic pain.

**Table 1:** **Summarized examples of impaired postural responses and associated cortical function**.

**Condition**	**Major reported impairments of postural responses**	**Cortical function during impaired standing postural responses**
Stroke involving cortex	Feet-in-place responses: change in the distal-to-proximal muscle pattern and delayed muscle onset as well as delayed and slowed rates of force development in the more severely involved limb (Di Fabio et al., 1986; Ikai et al., 2003)	Unaware of studies utilizing measures of cortical neurophysiology during standing postural responses post-stroke
	Stepping responses: paretic limb exhibits delayed foot-lift, low clearance, need for multiple steps, or an inability to initiate a step (Lakhani et al., 2011; Martinez et al., 2013). Steps are non-paretic limb dominant, even when obstacles block the limb (Lakhani et al., 2011; Mansfield et al., 2012). Impairments associate with increased fall risk and, in contrast, greater use of the paretic limb associates with better recovery (Mansfield et al., 2012, 2013)	
Advanced age	Age associates with delays and antagonistic co-contraction of muscles, as well as greater displacement and instability (Halicka et al., 2012)	Electroencephalographic (EEG) PEP delayed, bifid (double-peaked), and decreased with age—effects larger in balance impaired elders (Duckrow et al., 1999)
	Dual task costs greater for elderly only in late-phase in-place and stepping responses, more so for stepping and more so if balance impaired elder (Brown et al., 1999; Rankin et al., 2000; Brauer et al., 2001, 2002; Zettel et al., 2008)	
Parkinson's disease	Falls, instability, antagonistic co-contraction and lack of modulation based on knowledge of perturbation. Stepping responses may exhibit freezing or have decreased velocity and step length (Jacobs et al., 2005, 2009b; Jacobs and Horak, 2006; Smith et al., 2012)	Increased desynchronization of EEG beta signal, and this increase associates with decreased response adaptation to perturbation magnitude (Smith et al., 2012)
	With dual tasking, in-place responses are not modulated, and falls increase during stepping responses, but postural preparation or freezing unchanged (de Lima-Pardini et al., 2012; Jacobs et al., 2014a)	
Low back pain	Increased pre-perturbation muscle activation, increased activation amplitude at distal muscle, but decreased or delayed trunk muscle activation (Jacobs et al., 2011)	Late positive peak PEP larger with LBP; larger PEP correlates with less postural instability, disability, and fear of activity (Jacobs et al., 2014b)

### Stroke: insights into cortical neurophysiology of postural responses through cortical injury

Unfortunately, studies that measure cortical neurophysiology during standing postural responses of people with a history of stroke remain untested. Nevertheless, studies on stroke highlight the importance of the cortex for selecting and shaping postural response synergies and also highlight needs for understanding the neural mechanisms of altered postural responses following stroke.

Table 1 reinforces the importance of circuits involving the cortex for selecting an efficient and environmentally appropriate synergy (distal-to-proximal feet-in-place patterns and step selection in constrained conditions). The results also support a role for the cortex in generating the late-phase stepping response. Further, it is clear that mechanisms of both impairment and compensation exist, but such compensations (disuse of paretic limb) may represent increased fall risk and slowed recovery. A preliminary case study (Mansfield et al., 2011) suggests that compensatory step training can be beneficial, but such attempts at physical rehabilitation could be optimized if based in a more thorough understanding of the neural mechanisms responsible for these impairments and compensations. In addition, understanding neural mechanisms of impairment and compensation will better direct neurophysiologic treatments such as stimulation, pharmacology, or surgery. It is also unclear how the location and extent of post-stroke lesions affect different aspects of the postural response; the current literature does not clearly define lesion extent and includes injury of both cortical and subcortical regions. Thus, prognostics would benefit from being able to predict likely impairments from the lesion location and area. Clearly, too little is known about the effects of cortical injury on postural responses, and treatment options could be strongly influenced by a better understanding of cortical function during postural responses post-stroke.

### Aging: a lesson in how recording neurophysiology enhances knowledge beyond inference from behavior

Research on aging and cortical function during postural responses has largely been inferred through dual-task costs on the postural response under the assumption that a second task requires cortical resources and, therefore, any effects on postural responses reflect use of cortical resources during the response (Jacobs and Horak, 2007; Maki and McIlroy, 2007). As identified in Table 1, this assumption is compatible with findings that dual-task costs are most evident during the late phase of feet-in-place responses and the swing phase of stepping responses, which are thought to be cortically influenced (Jacobs and Horak, 2007). It remains unclear whether the age-related increase in dual-task costs represents greater compensation by cortical resources when sub-cortical processes of two tasks compete, or whether the cortex has greater influence on postural responses in any condition, which is revealed by competition for cortical resources when dual tasking. Knowing the difference would strongly influence interventions to target sub-cortical vs. cortical physiology as well as to determine the scope of circumstances in which impaired postural responses are a concern.

Recordings of cortical function during postural responses could provide key insight for identifying the mechanisms and circumstances of impairment, which could subsequently direct more optimized intervention. Unfortunately, only one study has attempted to compare EEG potentials evoked by perturbations of standing balance in young and older adults (Duckrow et al., 1999). Findings suggest that age associates with delayed, diminished and prolonged central sensory-motor processing at the cortex. This diminished and prolonged neural processing might explain the lack of a rapid and contextually optimized response. It remains unclear whether the cortex is the source of impairment or if its altered evoked potentials are subsequent to sub-cortical impairment, but the prolonged time to process the potentials at the cortex could explain the increased use of cortical resources suggested by dual-task studies and provides better focus for targets of further study and intervention that couldn't have been derived through only behavioral inference. Further study would benefit from high-resolution neurophysiologic recordings under single- vs. dual-task conditions in order to enhance understanding of the source and timing of impairments to ultimately direct more efficacious interventions.

### Parkinson's disease: a lesson in how recordings of cortical function reveal unexpected insights

In addition to the instability and falls associated with the impaired postural responses of people with PD, one of the more striking aspects is the lack of response modification based on contextual information about the upcoming perturbation's characteristics (Table 1). In addition, dual tasking during stepping responses does not alter early postural preparation, but does induce more falls, which suggests an ineffective step subsequent to the postural preparation (Jacobs et al., 2014a). The inability to optimize postural responses based on knowledge of perturbation characteristics and dual-task costs on only the late swing phase of stepping responses again suggest cortical involvement.

Because PD is often characterized as a disorder of diminished movement and cortical excitation during voluntary action, a contextually unmodified postural response might be predicted to associate with diminished preparatory potentials suggestive of a cortical incapacity to generate such potentials. Insights from EEG recordings of preparatory cortical function (contingent negative variation and event related desynchronization), however, demonstrate that people with PD fail to modulate their postural response by over-responding to small perturbations while concomitantly exhibiting increased desynchronization of upper beta (20–29 Hz) EEG signals prior to small perturbations. Further, larger desynchronization corresponds with less modulation of the postural response between small and large perturbation magnitudes (Smith et al., 2012). Beta desynchronization prior to movement is thought to represent motor preparation, inhibition of tonic activation, and/or anticipation of an impending need for movement within circuits that involve motor regions of the cortex (Jenkinson and Brown, 2011; Smith et al., 2012). Thus, recordings of cortical neurophysiology unexpectedly revealed that people with PD over-modulate their preparatory EEG activity prior to generating an unmodulated *hyper*metric response to small perturbations, rather than exhibiting diminished or unmodulated cortical preparation that coincides with the unmodulated response.

Given that preparatory cortical functions appear intact and over-responsive rather than incapacitated, these insights have significant ramifications for the potential to utilize behavioral and physical rehabilitation in order to train contextual response modulation. In addition, pharmacological or stimulation interventions would be directed differently for an over-responsive vs. under-responsive neurophysiologic condition. Therefore, this example in PD demonstrates the important value of recording cortical function during postural responses, because the neural mechanisms of a response may not be as expected based on behavioral inference alone.

### Chronic pain: demonstrating that cortical potentials to postural perturbations are functionally relevant

Chronic pain due to musculoskeletal injury such as LBP can significantly alter the central neural control of postural coordination. Although all phases of a postural response can be altered with LBP, the more consistent and significant findings are an enhanced muscle co-activation prior to perturbations and strongly diminished late-phase trunk muscle responses with concomitant increases in distal muscle responses (Table 1) (Jacobs et al., 2011). These pre-perturbation and late-phase alterations again implicate changes in cortical function during the postural response, and recent data demonstrate that late-phase evoked EEG potentials are enhanced with LBP. Interestingly, the enhancement appears compensatory because larger potential amplitudes correspond with less center-of-mass displacement, less disability, and less fear of physical activity (Jacobs et al., 2014b). Thus, these studies in LBP demonstrate two important lessons: (a) even peripheral musculoskeletal injuries alter the neural control of posture through the highest levels of the neural axis, and (b) cortical neurophysiology of postural responses can be relevant to the efficacy of the response and to clinical measures of disability.

## Summary

The examples above demonstrate that cortical neurophysiology during postural responses to extrinsic perturbations of standing balance is critical for individuals with postural impairments such as advanced aging, neurodegeneration, and chronic pain. In addition, the little available literature emphasizes how recordings of cortical neurophysiology during extrinsically induced postural responses can offer crucial insights into mechanisms of impaired balance that are not available or unexpected based on behavioral inference alone. Lastly, cortical neurophysiology is functionally relevant to the stability of postural responses and, perhaps, to clinical disability associated with the health condition.

Despite the importance of understanding the neurophysiologic mechanisms of impaired postural responses to extrinsic perturbations of standing balance, very little neurophysiologic recording beyond the muscle has been attempted during these responses. With technologies such as EEG, near infrared spectroscopy, single photon emission computed tomography, transcranial magnetic or direct-current stimulation, etc., and with improved abilities to overcome technical challenges, the opportunity for expansive research on the neurophysiology of extrinsically induced postural responses exists in order to compliment parallel work on voluntary postural control. For any population with disorders of balance and posture, more research is needed to evaluate multiple measures that reflect unique neurophysiologic systems of both preparatory and evoked neural activation. In addition, these recordings should be undertaken across multiple contexts that vary predictability, perturbation characteristics, dual tasking, etc. to more accurately understand how environmental circumstances affect the neural control of postural responses. In so doing, crucial insights are likely to emerge that could support more efficacious interventions and clinical outcomes for those with balance disorders.

### Conflict of interest statement

The author declares that the research was conducted in the absence of any commercial or financial relationships that could be construed as a potential conflict of interest.
